# THE TELEPHONE INTERVIEW FOR COGNITIVE STATUS (TICS): LATENT STRUCTURE OF COGNITION IN DOD-ADNI VIETNAM WAR VETERANS

**DOI:** 10.1093/geroni/igad104.1252

**Published:** 2023-12-21

**Authors:** Larry Tupler, Igor Akushevich

**Affiliations:** Duke University Medical Center, Durham, North Carolina, United States; Duke University, Durham, North Carolina, United States

## Abstract

The Telephone Interview for Cognitive Status (TICS) is a convenient, user-friendly neuropsychological tool developed to correlate with the MMSE and screen for dementia. Typically, thresholding supports diagnostic inferences despite the arbitrary assignment of individual item contributions to the score. Furthermore, although partially validated with World War II veterans, little subsequent psychometric work with aging military cohorts exists. We therefore deconstructed and analyzed the TICS dataset from the DoD-ADNI study recruitment for neuroimaging of Vietnam War veterans with traumatic brain injury (TBI) and/or posttraumatic stress disorder (PTSD) compared with deployed controls (N=1,828; mean age 69.4±4.5 years). Both disorders constitute putative risk factors for Alzheimer’s disease and related dementias. SAS statistics revealed raw and standardized Cronbach’s alpha with and without inclusion of missing values in the mid .70 range of internal-consistency reliability, with a dispersion of 0.55%–99.29% items incorrect. Exploratory factor analysis applying the Oblimin rotation of van den Berg et al. (2012; MEDLINE UI: 22384819) yielded results similar to that study of healthy civilians analyzing TICS section totals assigned a priori (e.g., temporal orientation, list learning). However, neuropsychological component processes were more subtly differentiated with our enrichment of analyses with deconstructed individual items, e.g., a factor structure reflecting primary versus secondary memory and speech-language. We discuss the advantage of differentiating this proposed latent structure of the TICS in achieving more precisely characterized neuropsychological domains of cognitive impairment underlying specific neurodegenerative and trauma-related dementias. We later apply these results to the longitudinal examination of cognitive aging, TBI, and PTSD in this cohort.

